# CDK5 Inhibits Synphilin-1 Ubiquitination and Basal Mitophagy: Implications for Parkinson’s Disease

**DOI:** 10.3390/ijms26168048

**Published:** 2025-08-20

**Authors:** Mor Savyon, Eyal Avraham, Ankit Kumar Shah, Haya Hamza, Raymonde Szargel, Fatimah Abd Elghani, Malik Farhoud, Michal Toren-Hershkoviz, Nicole Pavoncello, Sofia Zaer, Rina Bandopadhyay, Hazem Safory, Simone Engelender

**Affiliations:** 1 Department of Biochemistry, The B. Rappaport Faculty of Medicine and Institute of Medical Research, Technion-Israel Institute of Technology, Haifa 31096, Israel; morsavyon@gmail.com (M.S.); eyal.avr@gmail.com (E.A.); ankitkumarshah16@gmail.com (A.K.S.); hayahamzaa@gmail.com (H.H.); shargalr@g.technion.ac.il (R.S.); malik.far.96@gmail.com (M.F.); michal-her@campus.technion.ac.il (M.T.-H.); nicole.p@campus.technion.ac.il (N.P.); sofia.zair@campus.technion.ac.il (S.Z.); hsafory@technion.ac.il (H.S.); 2 Reta Lila Weston Institute of Neurological Studies, UCL Institute of Neurology, 1 Wakefield Street, London WC1N 1PJ, UK; rina.bandopadhyay@ucl.ac.uk

**Keywords:** Parkinson’s disease, neurodegeneration, synphilin-1, CDK5, ubiquitination, mitophagy, neuronal processes

## Abstract

Parkinson’s disease (PD) is characterized by the loss of dopaminergic neurons in the substantia nigra and the presence of α-synuclein-positive inclusions known as Lewy bodies. Synphilin-1 is a protein of unknown function that interacts with α-synuclein and has been shown to exhibit cytoprotective effects in both in vitro and in vivo models. In this study, we investigated whether synphilin-1 is phosphorylated by pathological CDK5 and explored the consequences of this modification. Pathological activation of CDK5 occurs mainly through its association with the calpain-cleaved protein p25. Although CDK5 inhibition protects against neurodegeneration in pharmacological PD models, we now show that p25 levels are increased in PD brains. Furthermore, we demonstrate that CDK5, in conjunction with p25, directly phosphorylates synphilin-1, mainly at serine 566. This phosphorylation reduces synphilin-1′s interaction with SIAH1, leading to reduced ubiquitination and subsequent accumulation. We also observed that CDK5-phosphorylated synphilin-1 exhibits a reduced ability to interact with PINK1 and to promote basal levels of mitophagy. Consistent with these findings, the phosphorylation-mimicking synphilin-1 S566E shows decreased translocation to mitochondria, and synphilin-1 levels are reduced in the mitochondria of PD brains compared to age-matched controls. Finally, synphilin-1 S566E promotes retraction of neuronal processes. Taken together, our results suggest that phosphorylation by CDK5 disrupts synphilin-1′s interactions with its protein partners, rendering it more toxic and impairing its ability to support mitophagy and maintain neuronal process homeostasis. We hypothesize that phosphorylation of synphilin-1 by CDK5 may contribute to the pathogenesis of PD.

## 1. Introduction

Parkinson’s disease (PD) is a common and incurable neurodegenerative disorder, characterized by the progressive loss of dopaminergic neurons in the substantia nigra and the presence of proteinaceous inclusions, known as Lewy bodies, in the surviving neurons [1]. α-Synuclein plays a central role in the pathogenesis of the disease, as the abnormal aggregation of this intrinsically disordered protein appears to initiate the formation of Lewy bodies in affected brains [2]. While several genes have been identified as mutated in familial forms of PD, including those encoding α-synuclein, LRRK2, PINK1, Parkin, DJ-1, VPS35, and GBA1, PD is believed to arise from a complex interplay between genetic and environmental factors, altogether disrupting cellular homeostasis [3,4]. In line with this, only about 10% of PD patients exhibit familial forms of the disease [4].

Synphilin-1 is a cytoplasmic protein encoded by the *SNCAIP* gene, which interacts with α-synuclein both in vitro and in neuronal tissue, and synphilin-1 is localized in Lewy bodies [5,6]. When expressed in cells, synphilin-1 forms cytosolic inclusions resembling Lewy bodies with α-synuclein [5,7], further supporting its involvement in PD. In mouse models, the expression of synphilin-1 together with α-synuclein disease mutations led to improved motor behavior and the formation of aggresome-like structures [8,9], consistent with findings that synphilin-1 aggregates are cytoprotective [10]. Notably, synphilin-1 was shown to reduce neuronal degeneration and facilitate the clearance of misfolded α-synuclein when co-expressed with α-synuclein A53T and A30P in mice, respectively [8,9].

The exact physiological function of synphilin-1 remains unclear. However, it has been associated with various cellular processes, including the modulation of synaptic vesicles, interaction with phospholipid membranes, cytoprotective activity, metabolic homeostasis, regulation of basal mitophagy rates, and modulation of cellular energy balance and energy homeostasis [10,11,12,13,14]. The effects of synphilin-1 expression in mice, however, are less well defined and range from hyperphagia and obesity to mild motor impairment [15,16].

Phosphorylation is a crucial post-translational modification (PTM) that regulates protein activity, signal transduction, and apoptosis [17,18,19]. It is also a prevalent PTM in Lewy bodies [20]. CDK5, a member of the cyclin-dependent kinase family, plays essential roles in the central nervous system, including brain development and the modulation of cognitive functions, such as synaptic plasticity, learning, and memory formation [21,22]. Under physiological conditions, CDK5 activity is regulated through its interaction with its protein partners, p35 and p39 [23,24].

CDK5 activity is elevated in several neurodegenerative diseases, particularly Alzheimer’s disease, where this increase is attributed to the accumulation of a truncated form of p35, known as p25 [25,26,27]. p25 is a carboxy-terminal fragment of p35, cleaved under toxic conditions by the cysteine protease calpain [28]. p25 binds directly to CDK5, leading to its redistribution within the cell and prolonged activation [26]. CDK5 has also been identified in Lewy bodies, suggesting its potential involvement in PD as well [29].

We report that endogenous p25 levels are elevated in the substantia nigra and frontal cortex of PD patients, supporting the notion that increased CDK5 activity may play a role in the pathogenesis of PD. We also demonstrate that synphilin-1 is a natural substrate of CDK5 and identify Ser566 as the primary phosphorylation site. Our findings show that CDK5-mediated phosphorylation at Ser566 inhibits synphilin-1 ubiquitination by SIAH1 and reduces its interaction with PINK1, as well as its translocation to the mitochondria. Consistent with this, both CDK5-phosphorylated synphilin-1 and the phosphorylation-mimicking synphilin-1 S566E exhibit a reduced ability to promote PINK1-dependent mitophagy. Additionally, we observe significantly decreased levels of synphilin-1 in mitochondrial fractions from PD brains. Furthermore, the phosphorylation-mimicking synphilin-1 S566E induces retraction of neuronal processes. These results suggest that CDK5 phosphorylation alters the intracellular targeting of synphilin-1, converting it into a toxic protein that fails to promote mitophagy. We propose that phosphorylation of synphilin-1 by CDK5 may contribute to the neurodegenerative processes underlying PD.

## 2. Results

**Figure 1:**
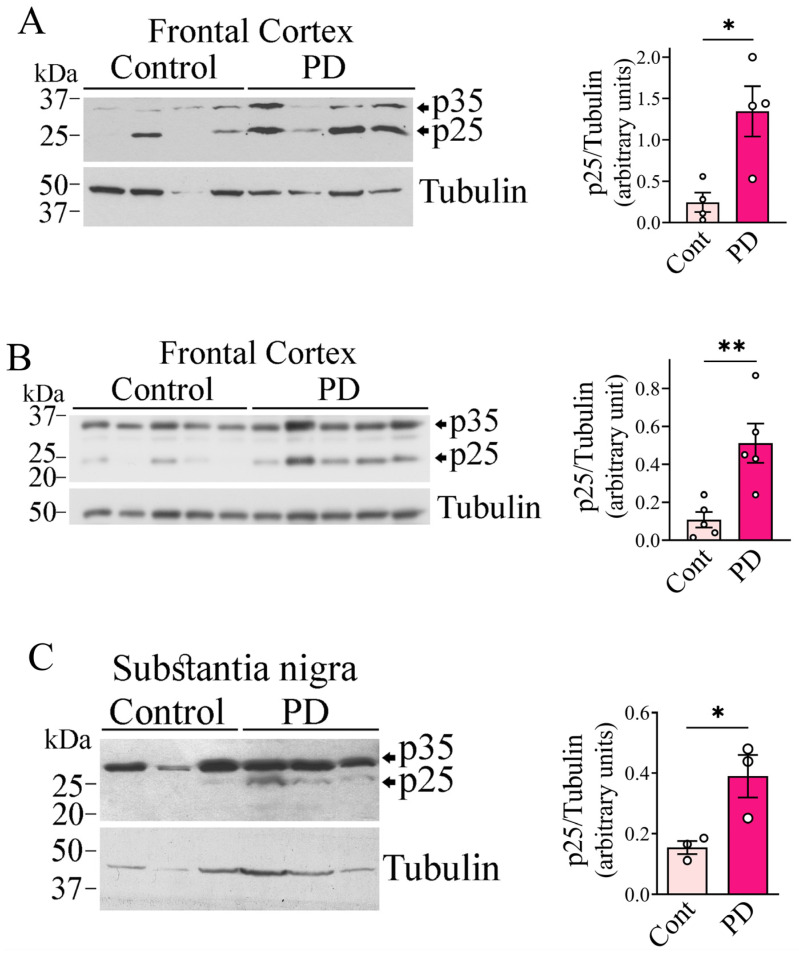
p25 levels are increased in the frontal cortex and substantia nigra in PD brains. (**A**,**B**), Homogenates from frontal cortex (**A**,**B**) and substantia nigra (**C**) of PD and matched controls were processed, and levels of p35/p25 were determined with anti-p35 antibody (upper panels). Graphs to the right depict the levels of p25 in cortex (**A**,**B**) and nigra (**C**) normalized to tubulin. Figures are representative of 3 independent Western blot analyses. Values represent the mean ± SEM of the samples analyzed in the corresponding blot (n = 4 (**A**), 5 (**B**), and 3 (**C**)). *, ** different from control at *p* = 0.04 (**A**), *p* = 0.0067 (**B**), and *p* = 0.0165 (Student’s *t*-test).

**P25 levels are increased in PD brains**. Synphilin-1 has previously been shown to be phosphorylated by GSK3β [18,30]. In this study, we sought to investigate whether CDK5 can also phosphorylate synphilin-1. CDK5 has been implicated in several neurodegenerative diseases, including AD, PD, and DLB [26,29,31]. Although it has been shown that knocking down CDK5 prevents dopaminergic degeneration in mice [31], the extent of CDK5 activation in PD remains unclear. We therefore examined the levels of p25, a known activator of CDK5, in brain tissues from PD patients. We homogenized the frontal cortex and substantia nigra from PD patients and age-matched controls, and quantified the levels of full-length p35 and its cleaved active product, p25, using an anti-p35 antibody. We found a significant increase in p25 levels in the frontal cortex of two different groups of PD patients (Figure 1A,B) compared to age-matched controls. Additionally, we observed up to a 4-fold increase in p25 levels in the substantia nigra of PD patients (Figure 1C), suggesting that CDK5 activity is likely elevated in PD.

**Figure 2:**
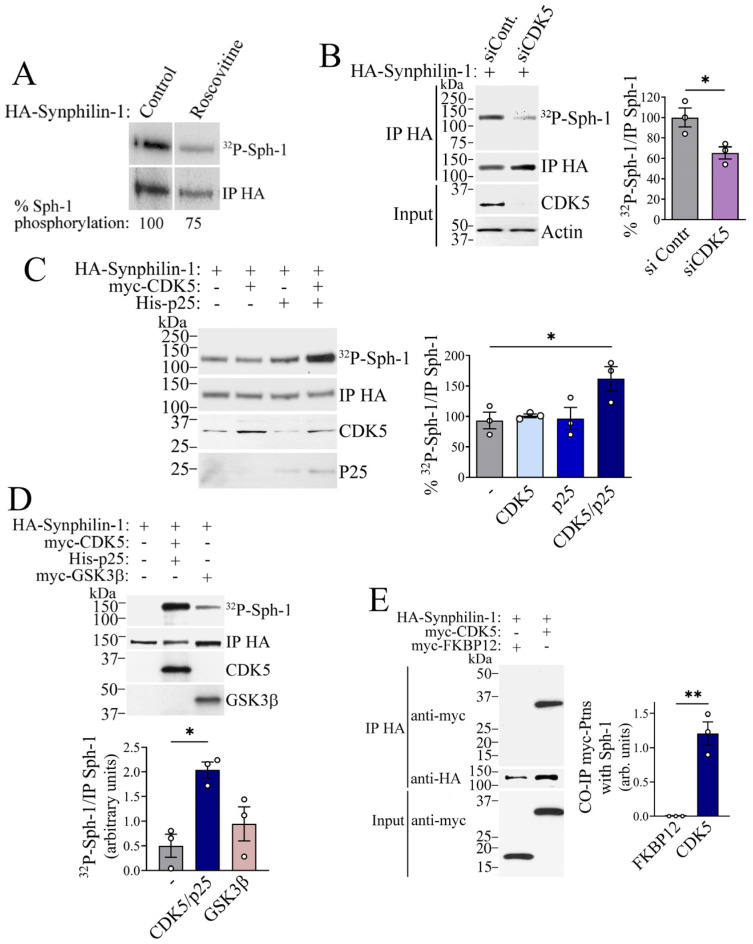
**CDK5 interacts and phosphorylates synphilin-1.** (**A**) Transfected HEK293 cells were incubated with ^32^P, in the absence and the presence of 1 μM roscovitine for 16 h. Phosphorylated synphilin-1 was immunoprecipitated with anti-HA and its levels determined by PhosphorImager analysis (upper panels). Levels of immunoprecipitated synphilin-1 were monitored using anti-HA (lower panels). (**B**) HEK293 cells were co-transfected with HA-synphilin-1 along with either control siRNA or siRNA to CDK5. Cells were incubated with ^32^P, and levels of phosphorylated synphilin-1 were determined as in A. Levels of CDK5 were determined with anti-CDK5 (third panel). The graph to the right represents the levels of phosphorylated synphilin-1 in the presence of siControl and siCDK5 relative to immunoprecipitated synphilin-1. Values represent the mean ± SEM of 3 independent experiments (n = 3). * different from control at *p* = 0.0342 (Student’s *t*-test). (**C**) HEK293 cells were transfected with HA-synphilin-1, in the absence or the presence of His-CDK5 and His-p25. Levels of phosphorylated and immunoprecipitated synphilin-1 were determined as in A. Levels of CDK5 and p25 were determined with anti-His (third panel and fourth panel, respectively). The graph to the right represents the levels of phosphorylated synphilin-1 in the absence and the presence of CDK5 and p25 relative to immunoprecipitated synphilin-1. Values represent the mean ± SEM of 3 independent experiments (n = 3). * different from control at *p* = 0.0369 (Repeated measures one-way ANOVA with Bonferroni post-hoc test). (**D**) Transfected HEK293 cells were processed, and levels of synphilin-1 phosphorylation were determined as described in A. Levels of CDK5 and GSK3β were determined with anti-His (third panel) and anti-myc (fourth panel), respectively. The graph below represents the levels of phosphorylated synphilin-1 in the presence of CDK5/p25 or GSK3β relative to immunoprecipitated synphilin-1. Values represent the mean ± SEM of 3 independent experiments (n = 3). * different from control at *p* = 0.0166 (Repeated measures one-way ANOVA with Bonferroni post hoc test). (**E**) Transfected HEK293 cells were lysed, and synphilin-1 was immunoprecipitated using anti-HA (second panel). Co-immunoprecipitation of interacting proteins was assessed using anti-myc (first panel). Input levels of myc-CDK5 and myc-FKBP12 were assessed using anti-myc antibody (third panel). The graph to the right represents the levels of myc proteins that co-immunoprecipitate with synphilin-1. Values represent the mean ± SEM of 3 independent experiments (n = 3). ** different from control at *p* = 0.0022 (Student’s *t*-test).

**CDK5 interacts with and phosphorylates synphilin-1**. To assess whether endogenous CDK5 phosphorylates synphilin-1, we transfected cells with HA-tagged synphilin-1 and labeled them with [^32^P] orthophosphate, in the absence and the presence of the CDK5 inhibitor roscovitine [32]. Phosphorylated synphilin-1 was immunoprecipitated using an anti-HA antibody, and its phosphorylation levels were quantified by PhosphorImager analysis. Treatment with roscovitine resulted in a significant reduction (~25%) in synphilin-1 phosphorylation, as indicated by the decreased [^32^P] incorporation in the immunoprecipitated protein (Figure 2A). Additionally, cells were co-transfected with synphilin-1 and either a control siRNA or CDK5-specific siRNA. Following [^32^P] labeling, the phosphorylation levels of synphilin-1 were assessed. CDK5 knockdown resulted in a marked decrease in synphilin-1 phosphorylation (Figure 2B), further supporting the role of CDK5 in mediating synphilin-1 phosphorylation. We next transfected cells with synphilin-1, either alone or in combination with CDK5 and its activator, p25 [26]. Co-expression of CDK5 and p25 increased synphilin-1 phosphorylation in cells (Figure 2C,D). Moreover, to compare the ability of CDK5 and GSK3β to phosphorylate synphilin-1, cells were transfected with synphilin-1 along with either CDK5 and p25 or GSK3β. [^32^P] incorporation was used to assess phosphorylation levels. We found that synphilin-1 phosphorylation by CDK5 was comparable to that induced by GSK3β (Figure 2D) [18]. Finally, to examine the interaction between synphilin-1 and CDK5, we co-expressed synphilin-1 with either CDK5 or the control FKBP12. Immunoprecipitation of synphilin-1 revealed that CDK5, but not FKBP12, co-immunoprecipitated with synphilin-1 (Figure 2E), providing further evidence that CDK5 interacts with and phosphorylates synphilin-1.

**Figure 3:**
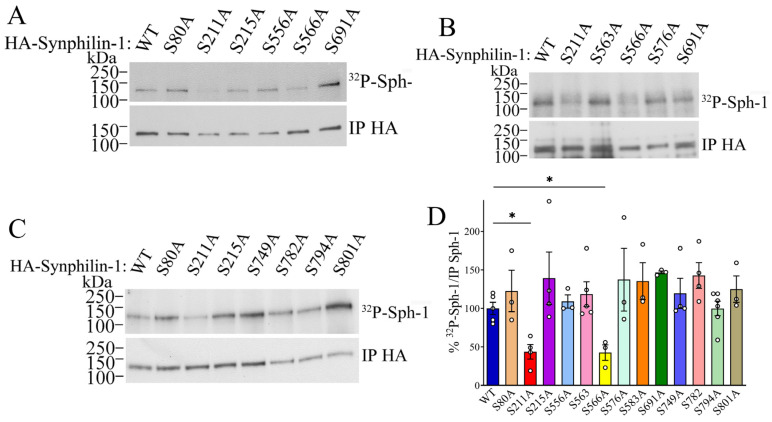
**Phosphorylation of synphilin-1 mutated at putative CDK5 sites.** (**A**–**C**) HEK293 cells were transfected with HA-synphilin-1 (wild-type or mutants) and incubated with ^32^P. Phosphorylated synphilin-1 was immunoprecipitated with anti-HA and levels of phosphorylation determined by PhosphorImager analysis (upper panels). Levels of immunoprecipitated synphilin-1 were determined with anti-HA (lower panels). (**D**) The graph represents the levels of phosphorylated synphilin-1 wild-type and mutants relative to HA-immunoprecipitated synphilin-1. Values represent the mean ± SEM of 3–6 independent experiments (n = 3–6). * different from control at *p* = 0.0307 (S211A) and 0.0423 (S566A) (repeated measures one-way ANOVA with Bonferroni post hoc test).

**CDK5 phosphorylates synphilin-1 at Ser566**. Synphilin-1 contains several putative CDK5 phosphorylation sites according to consensus motif (S/T)PX(K/R/H) [33], as well as an additional imperfect (S/T) P-derived motif that may also be an important target for CDK5 phosphorylation. Accordingly, only 49% of CDK5 substrates are phosphorylated at canonical motifs [34]. To identify the specific sites on synphilin-1 that are phosphorylated by CDK5, we employed two complementary approaches: mutagenesis and mass spectrometry analysis. First, we abrogated the putative CDK5 phosphorylation sites identified by the iGPS algorithm [35] by replacing the predicted serine/threonine residues with alanine. We next performed phosphorylation assays by transfecting cells with HA-tagged synphilin-1, either wild-type or mutant constructs. Following [^32^P] labeling, synphilin-1 was immunoprecipitated using anti-HA antibody. Among the putative CDK5 phosphorylation-deficient mutants, only the S211A and S566A mutants showed a significant reduction in phosphorylation levels, with decreases of 53% and 47%, respectively (Figure 3A–D). In contrast, other putative phosphorylation-deficient synphilin-1 mutants showed no significant change in phosphorylation levels compared to the wild-type protein (Figure 3A–D).

To confirm the specific sites phosphorylated by CDK5, we performed mass spectrometry analysis of immunoprecipitated synphilin-1 from cells expressing CDK5 and p25. Our analysis revealed that serine 566 in synphilin-1 is phosphorylated in the presence of CDK5 and p25, compared to the control in the absence of the enzyme (Figure S1). In contrast, serine 211 was phosphorylated both in the presence and absence of CDK5 and p25 (Figure S1). In addition to phosphorylation, deamidation was frequently detected in our mass spectrometry analysis (Figure S1). However, its levels did not differ between conditions with or without CDK5 and p25. Frequent detection of deamidation may reflect the high abundance of asparagine and glutamine residues in the synphilin-1 amino acid sequence, combined with the high sensitivity of the mass spectrometry method.

The mass spectrometry analysis also identified phosphorylation of CDK5-serine consensus sites S80 and S556. Still, these sites could not be confirmed by site-directed mutagenesis (Figure 3 and Figure S1). Additionally, other serine residues (S162, S167 and S674) were detected by mass spectrometry to be phosphorylated after the addition of CDK5 and p25 (S162, S167 and S674) (Figure S1), but they are not consensus for CDK5 phosphorylation [33], suggesting that they may be phosphorylated by other kinases regulated by CDK5 [36]. Since we could not confirm the phosphorylation of serines 80 and 211 through combined phosphorylation in cells and mass spectrometry analysis (Figure 3 and Figure S1), we chose to focus on serine 566 as the only pathologically relevant residue phosphorylated by CDK5. Consequently, we investigated the effects of synphilin-1 phosphorylation at serine 566 in subsequent experiments.

**Figure 4:**
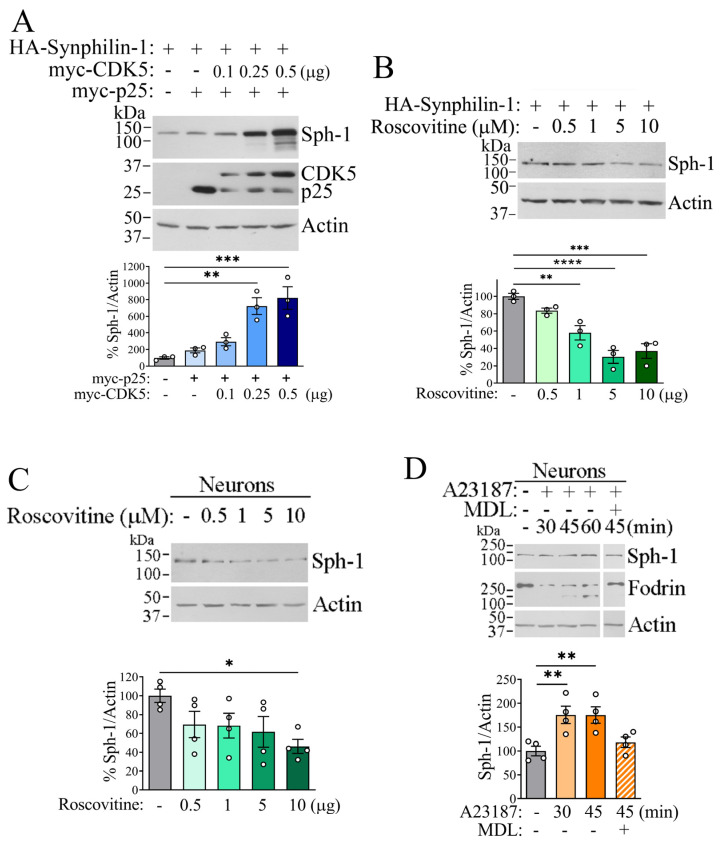
**Phosphorylation by CDK5 increases synphilin-1 steady-state levels.** (**A**) HEK293 cells were transfected with HA-synphilin-1, in the absence and the presence of myc-CDK5 and myc-p25. Total synphilin-1 levels were assessed using anti-HA antibody (first panel), while CDK5 and p25 levels were detected using anti-myc antibody (second panel). The graph below represents the levels of synphilin-1 in the presence of increasing CDK5 relative to actin. Values represent the mean ± SEM of 3 independent experiments (n = 3). **, *** different from control at *p* = 0.001 and 0.0003, respectively (repeated measures one-way ANOVA with Bonferroni post hoc test). (**B**) HA-synphilin-1 was transfected into HEK293 cells, which were then treated with increasing concentrations of roscovitine. Synphilin-1 expression was detected using anti-HA antibody (first panel). The graph below represents the levels of synphilin-1 in the presence of increasing roscovitine. Values represent the mean ± SEM of 3 independent experiments (n = 3). **, ***, **** different from control at *p* = 0.003, 0.0002 and <0.0001 (repeated measures one-way ANOVA with Bonferroni post hoc test). (**C**) Primary neurons were treated with increasing concentrations of roscovitine. Synphilin-1 levels were assessed using an anti-synphilin-1 antibody (first panel). The graph below represents the levels of synphilin-1 in the presence of increasing roscovitine. Values represent the mean ± SEM of 4 independent experiments (n = 4). * differentfrom control at *p* = 0.0281 (repeated measures one-way ANOVA with Bonferroni post hoc test). (**D**) Neurons were treated with 1 μM A23187 at different time points, in the absence or presence of 10 μM of the calpain inhibitor MDL-28170. Total levels of synphilin-1 were determined with anti-synphilin-1 (first panel). Levels of the calpain substrate fodrin were determined with anti-fodrin (second panel). The graph below represents the levels of synphilin-1 in the presence of A23187. Values represent the mean ± SEM of 4 independent experiments (n = 4). ** different from control at *p* = 0.0095 (30 min) and 0.0098 (45 min) (repeated measures one-way ANOVA with Bonferroni post hoc test).

**CDK5 regulates synphilin-1 steady-state levels by inhibiting its proteasomal degradation**. To explore how CDK5-mediated phosphorylation affects various properties of synphilin-1, we investigated its impact on synphilin-1 steady-state levels. HEK293 cells were co-transfected with synphilin-1, p25, and increasing amounts of CDK5. We observed a dose-dependent increase in synphilin-1 steady-state levels in response to CDK5 expression (Figure 4A). To confirm this effect, we treated synphilin-1-transfected cells with the CDK5 inhibitor roscovitine. Inhibition of CDK5 by roscovitine resulted in a reduction of synphilin-1 steady-state levels in both cells (Figure 4B) and neurons (Figure 4C). To further support these findings, we utilized the calcium ionophore A23187, which activates CDK5 by inducing calpain-dependent cleavage of p35 into p25 [28]. Neurons were treated with A23187 in the absence or presence of the calpain inhibitor MDL-28170. As expected, A23187 treatment increased the steady-state levels of synphilin-1 in neurons, an effect that was abolished by MDL-28170 (Figure 4D). These results further support the notion that CDK5-mediated phosphorylation promotes synphilin-1 accumulation in cells.

**Figure 5:**
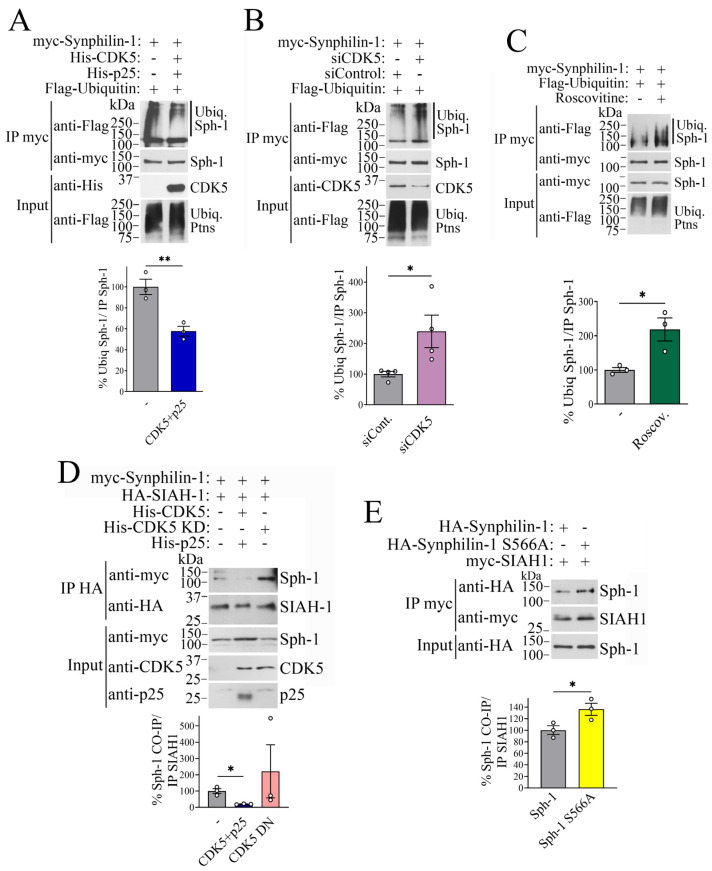
**Phosphorylation by CDK5 decreases synphilin-1 interaction with and ubiquitination by SIAH1.** (**A**) HEK293 cells were transfected with myc-synphilin-1, Flag-ubiquitin, in the absence and in the presence of His-CDK5 and His-p25. Synphilin-1 was immunoprecipitated with anti-myc (second panel), and levels of ubiquitinated immunoprecipitated synphilin-1 were determined with anti-Flag (first panel). Levels of CDK5 and total ubiquitinated proteins were determined with anti-CDK5 (third panel) and anti-Flag (fourth panel), respectively. The graph below represents the levels of ubiquitinated synphilin-1 in the absence and presence of CDK5/p25 relative to immunoprecipitated synphilin-1. Values represent the mean ± SEM of 3 independent experiments (n = 3). ** different from control at *p* = 0.0083 (Student’s *t*-test). (**B**) HEK293 cells were transfected with myc-synphilin-1, Flag-ubiquitin, in the presence of siRNA control or to CDK5. Cells were processed and analyzed as in A. The graph below represents the levels of ubiquitinated synphilin-1 in the presence of siControl or siCDK5 relative to immunoprecipitated synphilin-1. Values represent the mean ± SEM of 4 independent experiments (n = 4). * different from control at *p* = 0.0417 (Student’s *t*-test). (**C**) HEK293 cells were transfected with myc-synphilin-1 and Flag-ubiquitin, and treated with or without 1 μM roscovitine for 16 h. Cells were then processed and analyzed as described in A. The graph below shows the levels of ubiquitinated synphilin-1 in the absence or presence of roscovitine relative to immunoprecipitated synphilin-1. Values represent the mean ± SEM of 3 independent experiments (n = 3). * different from control at *p* = 0.0264 (Student’s *t*-test). (**D**) HEK293 cells were transfected with myc-synphilin-1, HA-SIAH1, in the absence or presence of His-CDK5, His-p25, and His-CDK5 dominant negative. SIAH1 was immunoprecipitated using anti-HA (second panel), and co-immunoprecipitated synphilin-1 was detected with anti-myc antibody (first panel). Total levels of synphilin-1 were assessed using anti-myc (third panel). Levels of CDK5 and p25 were determined with anti-CDK5 (fourth panel) and anti-p35 (fifth panel), respectively. The graph below represents the levels of synphilin-1 co-immunoprecipitation with SIAH1 in the absence and presence of CDK5 constructs. Values represent the mean ± SEM of 3 independent experiments (n = 3). * different from control at *p* = 0.0253 (Kruskal-Wallis test). (**E**) HEK293 cells were transfected with HA-synphilin-1 (wild-type or S566A) and myc-SIAH1. SIAH1 was immunoprecipitated using anti-myc (second panel), and co-immunoprecipitated synphilin-1 was detected with anti-HA antibody (first panel). Total levels of synphilin-1 were assessed using anti-HA (third panel). The graph below represents the levels of synphilin-1 co-immunoprecipitation with SIAH1. Values represent the mean ± SEM of 3 independent experiments (n = 3). * different from control at *p* = 0.0456 (Student’s *t*-test).

Since synphilin-1 is degraded by the ubiquitin-proteasome system [7], we investigated whether the accumulation of synphilin-1 by CDK5 is due to changes in its ubiquitination. To this end, cells were transfected with synphilin-1, either alone or together with CDK5 and p25. Synphilin-1 was immunoprecipitated, and levels of ubiquitinated synphilin-1 were assessed by Western blot analysis. Our results showed that CDK5 and p25 reduced the ubiquitination of synphilin-1 by approximately 40% (Figure 5A).

We next took the complementary approach of inhibiting CDK5, either through siRNA-mediated knockdown or pharmacological inhibition with roscovitine. Cells were transfected with synphilin-1 along with either control siRNA or CDK5-specific siRNA. CDK5 knockdown resulted in a ~2.5-fold increase in synphilin-1 ubiquitination (Figure 5B). Similarly, treatment of synphilin-1-expressing cells with roscovitine led to a comparable ~2.5-fold increase in ubiquitination levels (Figure 5C). These findings indicate that CDK5-mediated phosphorylation inhibits synphilin-1 ubiquitination, thereby stabilizing the protein by preventing its degradation via the ubiquitin-proteasome system.

SIAH1 is a RING-finger-containing E3-ubiquitin ligase that has been found in Lewy bodies [7], suggesting a potential role in PD pathogenesis. We previously demonstrated that SIAH1 interacts with synphilin-1, promotes its ubiquitination, and facilitates its proteasomal degradation [7,37]. To investigate whether CDK5-mediated phosphorylation affects this interaction, we co-transfected cells with synphilin-1 and SIAH1, in the absence or presence of CDK5 and p25, or a dominant-negative CDK5 mutant. SIAH1 was immunoprecipitated, and the extent of synphilin-1 co-immunoprecipitation was assessed. Our results show that CDK5 significantly reduces the interaction between synphilin-1 and SIAH1 (Figure 5D), whereas the dominant-negative CDK5 mutant had no effect on this interaction (Figure 5D). These findings suggest that the reduced interaction is dependent on CDK5 kinase activity and its ability to phosphorylate synphilin-1, rather than on the presence of CDK5 protein itself. To further validate these findings, we examined the interaction between SIAH1 and the CDK5 phosphorylation-deficient S566A mutant. Cells were co-transfected with SIAH1 and either wild-type synphilin-1 or the S566A mutant. We observed that the S566A mutant co-immunoprecipitated with SIAH1 more than the wild-type protein (Figure 5E), suggesting that phosphorylation of synphilin-1 at serine 566 by CDK5 reduces its interaction with SIAH1. Overall, our results indicate that CDK5-mediated phosphorylation of synphilin-1 modulates its interaction with SIAH1, leading to decreased ubiquitination and promoting its accumulation.

**Figure 6:**
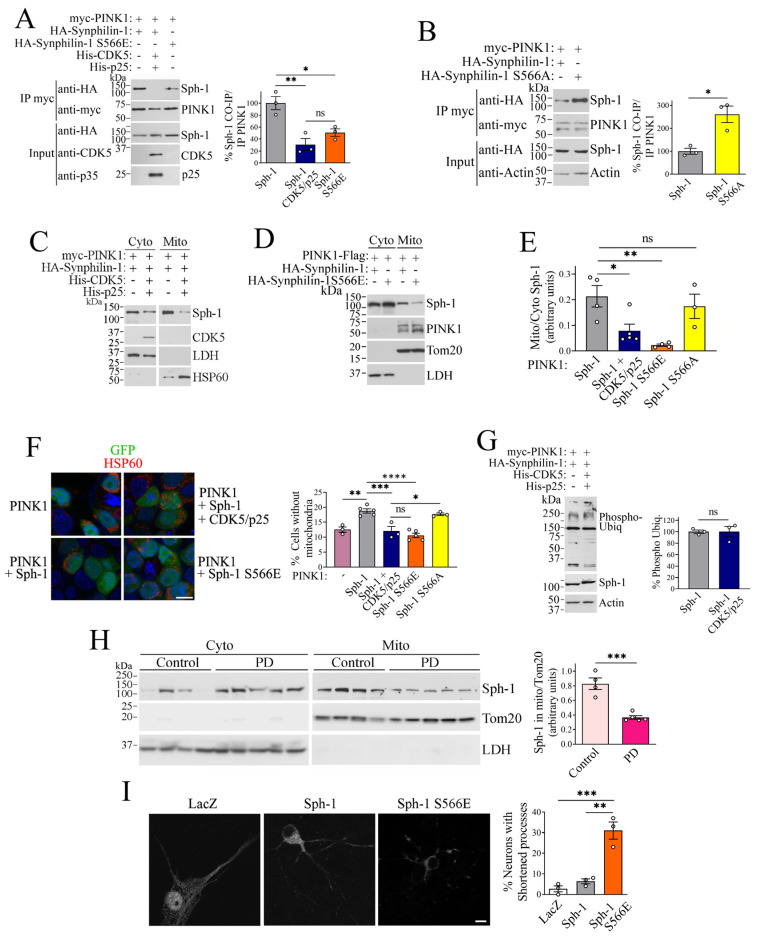
**Phosphorylation by CDK5 decreases synphilin-1 interaction with PINK1 and its ability to promote mitophagy.** (**A**) HEK293 cells were transfected with myc-PINK1, HA-synphilin-1 (wild-type or S566E), His-CDK5, and His-p25. PINK1 was immunoprecipitated with anti-myc (second panel), and synphilin-1 co-immunoprecipitation was identified using anti-HA (first panel). Total levels of synphilin-1 was determined with anti-HA (third panel). Levels of CDK5 and p25 were determined with anti-CDK5 (fourth panel) and anti-p35 (fifth panel), respectively. The graph to the right represents the levels of synphilin-1 co-immunoprecipitation with PINK1 in the absence and presence of CDK5 and p25. Values represent the mean ± SEM of 3 independent experiments (n = 3). *, ** different from control at *p* = 0.0362 and 0.0075, respectively (repeated measures one-way ANOVA with Bonferroni post hoc test). (**B**) HEK293 cells were transfected with myc-PINK1 and HA-synphilin-1 (wild-type or S566A). Lysates of transfected cells were immunoprecipitated and analyzed as in A. The graph to the right depicts the levels of synphilin-1 co-immunoprecipitation with PINK1. Values represent the mean ± SEM of 3 independent experiments (n = 3). * different from control at *p* = 0.0415 (Student’s *t*-test). (**C**) HEK293 cells were transfected with myc-PINK1 and HA-synphilin-1, in the absence or presence of His-CDK5 and His-p25. Synphilin-1 distribution between cytosolic and mitochondrial fractions was determined with anti-HA (first panel). The purity fractions were determined by the levels of HSP60 and LDH (third and fourth panels). (**D**) HEK293 cells were transfected with PINK1-Flag and HA-synphilin-1 (wild-type or S566E). The distribution of synphilin-1 across the fractions was assessed as described in C. (**E**) HEK293 cells were transfected with myc-PINK1 and HA-synphilin-1 (wild-type, S566E or S566A), in the absence or presence of His-CDK5 and His-p25. The graph shows the relative levels of synphilin-1 in mitochondrial fractions (normalized to HSP60 or Tom20) compared to cytosolic fractions (normalized to LDH). Values represent the mean ± SEM of 3–5 experiments (n = 3–5). *, ** different from control at *p* = 0.0215 and 0.003, respectively. NS, *p* > 0.9999 (repeated measures one-way ANOVA with Bonferroni post hoc test). (**F**) HEK293 cells were transfected with GFP, PINK1-Flag, HA-synphilin-1 (wild-type, S566E or S566A), in the absence or presence of His-CDK5 and His-p25. Cells were processed for immunocytochemistry with anti-HSP60, and analyzed by confocal microscopy. The graph to the right represents the percentage of transfected cells (green) that lack mitochondria (HSP60, red). Values represent the mean ± SEM of 3–5 independent experiments (n = 3–5). *, **, ***, **** different from control at *p* = 0.0114, 0.0017, 0.0008 and < 0.0001, respectively. NS, *p* > 0.9999 (repeated measures one-way ANOVA with Bonferroni post hoc test). (**G**) HEK293 cells were transfected with PINK1-Flag, HA-synphilin-1, in the absence or presence of His-CDK5 and His-p25. Cells were treated with 10 μM CCCP. Total levels of phosphorylated ubiquitin were determined with anti-phospho-ubiquitin (first panel). The graph to the right represents the percentage of phosphorylated ubiquitin in the absence and presence of CDK5/p25 relative to actin. Values represent the mean ± SEM of 3 independent experiments (n = 3). NS, *p* = 0.931 (Student’s *t*-test). (**H**) Cytosolic and mitochondrial fractions isolated from the frontal cortex of PD and matched controls were analyzed for synphilin-1 levels using anti-synphilin-1 antibody (upper panels). Purity of cytosolic and mitochondrial fractions was determined with anti-LDH (lower panels) and anti-Tom20 (middle panels), respectively. The graph to the right depicts the levels of synphilin-1 relative to Tom20 in mitochondrial fractions. The figure is representative of 3 independent sample processing and Western blot analyses. Values represent the mean ± SEM of analyzed samples in the shown blot (n = 4 and 5). *** different from control at *p* = 0.0004 (Student’s *t*-test). (**I**) HA-synphilin-1 (wild-type or S566E) or HA-LacZ was transfected into primary neurons. Neurons were processed for immunocytochemistry with anti-HA and analyzed by confocal microscopy. Scale, 10 μm. The graph to the right represents the percentage of transfected neurons with processes equal to or shorter than 25 μμm. Values represent the mean ± SEM of 3 experiments. **, *** different from controls at *p* = 0.0017 and 0.0008 (repeated measures one-way ANOVA with Bonferroni post hoc test).

**Subcellular localization of synphilin-1 is regulated by CDK5 phosphorylation**. We have previously shown that synphilin-1 is recruited to mitochondria by PINK1, triggering mitochondrial depolarization and mitophagy in a Parkin-independent, SIAH1-dependent process [12]. In this study, we investigated whether phosphorylation by CDK5 impairs synphilin-1 translocation to the mitochondria and its role in promoting mitophagy. We first examined the impact of CDK5-mediated phosphorylation on the interaction between synphilin-1 and PINK1. Cells were transfected with PINK1 and either wild-type synphilin-1 or the phosphorylation-mimicking mutant, in the absence or presence of CDK5 and p25. Co-immunoprecipitation analysis revealed that CDK5 and p25 co-expression reduced the interaction between synphilin-1 and PINK1 by approximately 65% (Figure 6A). Similarly, the S566E mutant exhibited a diminished ability to co-immunoprecipitate with PINK1 (Figure 6A), consistent with phosphorylation at serine 566 impairing their interaction. Conversely, the phosphorylation-deficient S566A mutant co-immunoprecipitated with PINK1 to a greater extent than wild-type synphilin-1 (Figure 6B).

Since mitochondrial translocation of synphilin-1 depends on its interaction with PINK1 [12], we next investigated whether CDK5-mediated phosphorylation influences this process. For this, we transfected cells with PINK1 and synphilin-1 (wild-type or S566 mutants), in the absence and presence of CDK5 and p25, and tracked the translocation of synphilin-1 proteins to the mitochondria. We found that CDK5 and p25 expression reduced the presence of synphilin-1 in mitochondrial fractions by approximately 60% (Figure 6C,E). Similarly, the phosphorylation-mimicking mutant S566E showed an 80% reduction in mitochondrial localization compared to wild-type synphilin-1 (Figure 6D,E), suggesting that CDK5-mediated phosphorylation negatively regulates synphilin-1 translocation to the mitochondria. In contrast, the phosphorylation-deficient mutant S566A showed no significant difference in mitochondrial localization related to the wild-type protein (Figure 6E), implying that additional factors may influence PINK1 availability on the outer mitochondrial membrane.

**Phosphorylation of synphilin-1 by CDK5 impairs its role in supporting basal mitophagy**. We next examined the impact of phosphorylated synphilin-1 on mitophagy. As previously reported [12], co-expression of synphilin-1 and PINK1 resulted in a greater reduction in mitochondrial content compared to PINK1 alone (Figure 6F). However, when CDK5 and p25 were co-expressed with synphilin-1 and PINK1, this reduction was significantly attenuated, indicating impaired mitophagy (Figure 6F). Similarly, the phosphorylation-mimicking S566E mutant of synphilin-1 exhibited a diminished capacity to reduce mitochondrial content compared to the wild-type protein (Figure 6F). In contrast, the phosphorylation-deficient S566A mutant did not further enhance mitophagy compared to wild-type synphilin-1 (Figure 6F), consistent with its inability to translocate more to the mitochondria than the wild-type synphilin-1 (Figure 6E), further underscoring the importance of synphilin-1 mitochondrial localization to promote mitophagy. Together, these findings indicate that CDK5-mediated phosphorylation of synphilin-1 impairs its mitochondrial targeting and, consequently, its ability to promote mitophagy.

The PINK1-Parkin pathway promotes mitophagy through activation of PINK1 kinase activity and phosphorylation of ubiquitin [38,39,40]. Although the PINK1-synphilin-1 pathway functions independently of PINK1 kinase activity [12], we investigated whether CDK5 might exert unexpected effects on PINK1 that could also influence the PINK1-synphilin-1 pathway. To address this, cells co-transfected with PINK1 and synphilin-1, in the absence or presence of CDK5 and p25, were treated with the mitochondrial depolarizing agent CCCP to induce PINK1-mediated ubiquitin phosphorylation. We observed no change in phosphorylated ubiquitin levels in the presence of CDK5 and p25 (Figure 6G), suggesting that the observed reduction in mitophagy is likely due to CDK5-mediated phosphorylation of synphilin-1, rather than altered PINK1 activity.

Next, we examined whether synphilin-1 mitochondrial translocation is altered in PD. Cytosolic and mitochondrial fractions were isolated from the frontal cortex of PD and age-matched controls, and synphilin-1 levels were analyzed. We observed a significant reduction in synphilin-1 levels within the mitochondrial fractions of PD samples compared to controls (Figure 6H). This finding is consistent with our results showing that CDK5 and p25 impair synphilin-1 mitochondrial translocation (Figure 6C–E). It also aligns with the elevated p25 levels in PD brains (Figure 1) and supports previous evidence implicating CDK5 activity in dopaminergic neuronal degeneration in PD mouse models [31].

Synphilin-1 has been shown to be cytoprotective [10] and is known to translocate not only to mitochondria but also to synaptic terminals [12,41]. Within this context, we investigated whether CDK5-mediated phosphorylation alters the cytoprotective properties of synphilin-1 in neurons. Given that CDK5 and p25 expression are known to induce neuronal process retraction on their own [26], we examined the effect of the phosphorylation-mimicking synphilin-1 S566E mutant. We transfected neurons with wild-type synphilin-1 or the phosphorylation-mimicking S566E mutant. Approximately 30% of neurons expressing the S566E mutant exhibited neurite retraction, in contrast to those expressing wild-type synphilin-1 or the control protein LacZ (Figure 6I). These findings suggest that CDK5-mediated phosphorylation converts synphilin-1 into a neurotoxic protein.

Taken together, our results suggest that phosphorylation of synphilin-1 by pathologically activated CDK5 reduces its translocation to mitochondria, resulting in the conversion of synphilin-1 into a toxic protein unable to contribute to mitophagy.

## 3. Discussion

Synphilin-1 is an α-synuclein-interacting protein whose function remains unclear, but it is present in Lewy bodies [5,6]. A mutation in synphilin-1 (R621C) has been identified in two German PD patients [42]. While this mutation may highlight the potential role of synphilin-1 in the disease, it has not been detected in other populations [43]. In this study, we propose that post-translational modifications could render synphilin-1 toxic to neurons in PD. Specifically, we demonstrate that synphilin-1 is a substrate of CDK5. Using site-directed mutagenesis and mass spectrometry, we identified serine 566 as the site of phosphorylation by CDK5 in the presence of p25. This phosphorylation decreases synphilin-1′s ability to interact with SIAH1, which in turn reduces its ubiquitination and proteasomal degradation. Consequently, CDK5-phosphorylated synphilin-1 exhibits an extended half-life, leading to its accumulation in cells and neurons. Furthermore, CDK5 phosphorylation impairs the interaction between synphilin-1 and PINK1, reducing its ability to translocate to mitochondria and support mitophagy via the PINK1-synphilin-1 pathway. In line with this, the phosphorylation-mimicking mutant of synphilin-1 (S566E) shows decreased mitochondrial translocation and diminished mitophagy-promoting activity. Most importantly, we observed that PD brains accumulate p25, exhibit reduced synphilin-1 levels in mitochondria, and show evidence that CDK5-phosphorylated synphilin-1 contributes to the retraction of neuronal processes. These findings suggest that CDK5 phosphorylation alters synphilin-1 the intracellular distribution of synphilin-1, impairs its role in basal mitophagy, and disrupts the maintenance of neuronal processes, potentially contributing to neuronal dysfunction in PD.

We found that endogenous CDK5 phosphorylates synphilin-1, as evidenced by reduced synphilin-1 phosphorylation following treatment with roscovitine or CDK5-targeting siRNA. However, the primary focus of this study was to investigate the pathological role of CDK5 in PD. To that end, we conducted most of the experiments using either a dominant-negative CDK5 construct or synphilin-1 S566 mutants to specifically assess the deleterious effects of pathologically activated CDK5. Among the predicted CDK5 phosphorylation sites in synphilin-1, we identified serine 566 as the principal residue phosphorylated under pathological conditions. Accordingly, our investigation centered on serine 566 to elucidate its role in PD-related mechanisms. Nonetheless, additional studies will be necessary to determine the physiological significance of CDK5-mediated phosphorylation of synphilin-1 under normal conditions.

We have shown that the phosphorylation-mimicking synphilin-1 S566E mutant has a reduced ability to interact with PINK1, resulting in decreased translocation to the mitochondria and an inability to promote mitophagy through the PINK1-synphilin-1 pathway. In contrast, the phosphorylation-deficient S566A, while interacting more effectively with PINK1, does not exhibit an increased ability to translocate to the mitochondria or enhance mitophagy. The underlying reason for this is unclear, but it is possible that additional factors influence the mitochondrial translocation of synphilin-1. For instance, the availability of free PINK1 at the mitochondrial outer membrane may be limited, or other proteins may be required to anchor synphilin-1 at the membrane. Further research is needed to uncover the mechanisms that regulate synphilin-1 localization to the mitochondrial membrane, which could offer new insights into strategies for enhancing mitochondrial renewal in PD.

Previous studies have shown that the expression of CDK5 and p25 induces retraction of neuronal processes in culture [26]. In this study, we found that synphilin-1 phosphorylation-mimicking S566E mutant also leads to similar retraction of neuronal processes. This observation supports the notion that CDK5-mediated phosphorylation may shift synphilin-1 from a cytoprotective role [10,42] to a more neurotoxic one. However, further studies are needed to determine whether the toxic effects of CDK5-phosphorylated synphilin-1 are due to its impaired ability to promote basal mitophagy or whether additional changes in its properties contribute to this neurotoxicity.

Synphilin-1 has been previously shown to be phosphorylated by GSK3β [18]. Drawing a parallel to the activation of GSK3β and CDK5 in neurodegenerative diseases, particularly the hyperphosphorylation of tau in AD [44], it is plausible that synphilin-1 may also undergo hyperphosphorylation in PD. Supporting this possibility, we observed evidence of CDK5 activation in PD, as indicated by elevated levels of its activator p25 in patient samples. However, given the limited sample size, analysis of additional patient tissues will be important to strengthen this observation. Furthermore, reduced protein yield in some of the analyzed samples suggests suboptimal preservation in certain post-mortem tissues. Importantly, it remains unclear whether CDK5-mediated phosphorylation of synphilin-1 is elevated in the brains of patients with PD. Future studies employing antibodies specific to CDK5-phosphorylated synphilin-1 will be necessary to accurately determine the extent of this modification in PD brain tissues.

While hyperphosphorylation of tau by GSK3β and CDK5 promotes its aggregation [45], further studies are needed to determine whether CDK5 and GSK3β also drive the aggregation of synphilin-1, and how this may impact the aggregation of α-synuclein. We have previously demonstrated that inhibition of GSK3β enhances the ubiquitination of synphilin-1 and promotes the formation of ubiquitinated inclusions under conditions of proteasomal inhibition [18]. However, the role of phosphorylated synphilin-1 in inclusion formation in PD remains to be fully understood and warrants further investigation.

CDK5 is implicated in several neurodegenerative diseases [19,46]. In AD, early studies demonstrated that increased cleavage of p35 into p25 leads to pathological hyperphosphorylation of tau [26,46]. Similarly, in PD, numerous studies suggest that CDK5 activation may contribute to neuronal loss. For example, apoptosis induction in the substantia nigra of rats results in elevated CDK5 expression [47], and CDK5 is found in Lewy bodies [29]. Additionally, general CDK5 inhibitors and the expression of dominant-negative CDK5 reduce dopaminergic neuron loss in MPTP-treated mice [31]. However, to our knowledge, we are the first to directly demonstrate elevated p25 levels in PD brains, further supporting the role of CDK5 activation in PD. These findings suggest that inhibition of CDK5 activity using the small, brain-penetrant CDK5 inhibitor peptide CDK5i [48] could provide a promising therapeutic strategy for PD.

Since α-synuclein lacks consensus CDK5 phosphorylation sites, other CDK5 substrates are likely contributing to the toxicity associated with CDK5 activation in PD. For example, CDK5-mediated phosphorylation of the antioxidant enzyme Prx2 reduces its activity, thereby increasing neuronal vulnerability to mitochondrial stress [49]. Additionally, CDK5 phosphorylation inhibits the ubiquitin ligase activity of Parkin, potentially impairing its ability to promote mitophagy of damaged mitochondria [50]. Building on these findings, we propose that increased phosphorylation of synphilin-1 may also contribute to the pathological effects of CDK5 activation. In this context, targeting CDK5-mediated phosphorylation of synphilin-1 could represent a novel therapeutic approach for PD.

Our results suggest that synphilin-1 is specifically phosphorylated at serine 566 by the CDK5/p25, a modification that impairs its ability to promote basal mitophagy and maintain the integrity of neuronal processes. The phosphorylation of synphilin-1 by CDK5 may have significant implications for the pathogenesis of PD.

## 4. Materials and Methods

**Cell Cultures and Transfections**. Cells were cultured in Dulbecco’s Modified Eagle Medium (DMEM) containing 10% fetal bovine serum, under a 5% carbon dioxide (CO_2_) atmosphere. HEK293 cells were transiently transfected with N- or C-terminal-tagged pRK5 plasmids using Lipofectamine 2000 (ThermoFischer Scientific, Waltham, MA, USA) and harvested 36 hours post-transfection. For siRNA experiments, cells were transfected with siCDK5 (ID: 1466) or a scrambled siRNA control (Ambion, Waltham, MA, USA) using Lipofectamine 2000, as previously described [18].

**Western Blot Analysis**. Samples were homogenized in buffer containing 50 mM Tris (pH 7.4), 140 mM NaCl, 1% Triton X-100, 0.1% SDS, 30 μM MG132, and a protease inhibitor cocktail (MiniComplete, Roche, Basel, Switzerland). Cell lysates were clarified by centrifugation at 13,000× *g* for 5 min. Proteins were separated by 10% SDS-PAGE and transferred to nitrocellulose membranes. Membranes were probed with the following primary antibodies: mouse anti-HA (Covance, MMS-101, Princeton, NJ, USA), mouse anti-actin (MP Biomedicals, 691001, Santa Ana, CA, USA), mouse and rabbit anti-myc, rabbit anti-HA, rabbit anti-lactate dehydrogenase, mouse anti-HSP60, mouse anti-ubiquitin (Santa Cruz, sc-789, sc-805, sc-33781, sc-11415, sc-8017), mouse anti-myc (Sigma, M4439, Burlington, MA, USA), rabbit anti-Flag (Sigma, F7425), and rabbit anti-phopho-ubiquitin (Boston Biochem A-110, Cambridge, MA, USA). Signal detection was performed using enhanced chemiluminescence (ECL Dura, ThermoFischer Scientific), and quantification was carried out using ImageMaster analysis (GE Healthcare Life Sciences, Waukesha, WI, USA). 

**Co-immunoprecipitation Assays**. To examine the interaction between synphilin-1 and PINK1, co-immunoprecipitation was performed using lysates from transfected cells. Cells were lysed in buffer containing 50 mM Tris-HCl (pH 7.4), 140 mM NaCl, 1% Triton X-100, 0.1% SDS, 30 μM MG132, 20 mM NaF, 2 mM Na_3_VO_4_, 10 mM PPi, 20 mM β-glycerol phosphate, and a protease inhibitor cocktail (MiniComplete, Roche, Basel, Switzerland). Lysates were clarified by centrifugation at 13,000× *g* for 5 min, followed by incubation with anti-HA antibody conjugated to protein G agarose beads (Sigma, Burlington, MA, USA) for 4 h at 4 °C. Immunoprecipitates were washed with lysis buffer containing 500 mM NaCl and detected by Western blot analysis.

**In Vitro Phosphorylation Assays**. HEK293 cells were transfected with HA-synphilin-1 cDNA. Thirty-six hours after transfection, cells were lysed as described above for the co-immunoprecipitation experiments. HA-synphilin-1 was immunoprecipitated with anti-HA antibody and washed with lysis buffer containing 500 mM NaCl. Immunoprecipitated synphilin-1 was incubated with recombinant CDK5/p25 (Upstate) at 37 °C for 1 h in buffer containing 40 mM Tris (pH 7.6), 2 mM dithiothreitol, 5 mM MgCl2, 2 μg/mL soybean trypsin inhibitor, 0.05 mM unlabeled ATP, and 0.25 mCi/mL [γ-^32^P] ATP. Reactions were stopped by adding SDS-sample buffer and analyzed by SDS-PAGE. The amount of ^32^P-labeled synphilin-1 was quantified by PhosphorImager analysis. Loading of immunoprecipitated HA-synphilin-1 was determined by Western blot using anti-HA antibody.

**In Vivo Phosphorylation Assays**. After overnight incubation in phosphate-free medium, transfected HEK293 cells were incubated for 4 h at 37 °C with serum-free/phosphate-free medium containing 200–400 μCi/mL [^32^P]orthophosphate. Cells were harvested and lysed in buffer containing 50 mM Tris-HCl (pH 7.4), 140 mM NaCl, 1% Triton X-100, 0.1% SDS, 20 mM NaF, 2 mM Na_3_VO_4_, 10 mM PPi, 20 mM β-glycerol phosphate, 30 μM MG132 and protease inhibitor mixture (MiniComplete, Roche). Immunoprecipitation of HA-synphilin1-1 was carried out with anti-HA antibody for 4 h at 4 °C. Beads were washed with lysis buffer supplemented with 500 mM NaCl and analyzed by 10% SDS-PAGE. Densitometric quantification of radiolabeled HA-synphilin-1 was carried out by PhosphorImager analysis. Loading of immunoprecipitated HA-synphilin-1 was determined by Western blot using anti-HA antibody.

**Mass Spectrometry Analysis**. HEK293 cells were transfected with HA-synphilin-1, in the absence and in the presence of CDK5 and p25. After 48 h of transfection, cells were lysed in buffer containing 50 mM Tris (pH 7.4), 140 mM NaCl, 1% Triton X-100, 0.1% SDS, 20 mM NaF, 2 mM Na_3_VO_4_, 10 mM PPi, 20 mM, β-glycerol phosphate, 10 μM lactacystin, and protease inhibitor mixture (MiniComplete, Roche). Immunoprecipitation of HA-synphilin1-1 was carried out with anti-HA antibody for 4 h at 4 °C. Immunoprecipitates were washed with lysis buffer containing 500 mM NaCl. Samples were run on SDS-PAGE, processed for Coomassie blue staining, and the immunoprecipitated synphilin-1 band was cut for mass spectrometry analysis (Proteomic and Metabolomic Core Facility, Duke University). Briefly, Coomassie-stained SDS-PAGE bands were subjected to standardized in-gel trypsin digestion in which gel bands were subjected to reduction with 10 mM dithiothreitol, alkylated with 20 mM iodoacetamide, and digested with 100 ng of sequencing grade modified trypsin (Promega). Extracted peptides were lyophilized to dryness and resuspended in 12 uL of 0.2% formic acid/2% acetonitrile. For phosphopeptide-enriched samples, digested peptides were resuspended in 80% acetonitrile/1% TFA and subjected to TiO_2_ enrichment on GL Sciences tips according to manufacturer recommendations. Each sample was subjected to chromatographic separation on a Waters NanoAquity UPLC equipped with a 1.7 µm BEH130 C_18_ 75 µm I.D. X 250 mm reversed-phase column. The mobile phase consisted of (A) 0.1% formic acid in water and (B) 0.1% formic acid in acetonitrile. Following a 3 µL injection, peptides were trapped for 3 min on a 5 µm Symmetry C_18_ 180 µm I.D. X 20 mm column at 5 µL/min in 99.9% A. The analytical column was then switched in-line, and a linear elution gradient of 5% B to 40% B was performed over 60 min at 400 nL/min. The analytical column was connected to a fused silica PicoTip emitter (New Objective, Cambridge, MA, USA) with a 10 µm tip orifice and coupled to a QExactive Plus mass spectrometer (Thermo) through an electrospray interface operating in a data-dependent mode of acquisition. The instrument was set to acquire a precursor MS scan from *m/z* 375–1675 with MS/MS spectra acquired for the ten most abundant precursor ions. For all experiments, HCD energy settings were 27 v and a 120 s dynamic exclusion was employed for previously fragmented precursor ions.

Raw LC-MS/MS data files were processed in Proteome Discoverer (Thermo Scientific) and then submitted to independent Mascot searches (Matrix Science) against a SwissProt database (*Human* taxonomy) containing both forward and reverse entries of each protein (20,322 forward entries). Search tolerances were 5 ppm for precursor ions and 0.02 Da for product ions using trypsin specificity with up to two missed cleavages. Carbamidomethylation (+57.0214 Da on C) was set as a fixed modification, whereas oxidation (+15.9949 Da on M), deamidation (+0.98 Da on NQ), and phosphorylation (+79.998 Da Ser/Thr/Tyr) were considered dynamic mass modifications. All searched spectra were imported into Scaffold (v4.3, Proteome Software; http://www.proteomesoftware.com), and scoring thresholds were set to achieve a peptide false discovery rate of 1% using the PeptideProphet algorithm.

**In Vivo Ubiquitination Assays**. Transfected HEK293 cells were incubated with 10 μM lactacystin for 12 h and then lysed in buffer containing 50 mM Tris (pH 7.4), 140 mM NaCl, 1% Triton X-100, 0.1% SDS, 20 mM NaF, 2 mM Na_3_VO_4_, 10 mM PPi, 20 mM, β-glycerol phosphate, 10 μM lactacystin and protease inhibitor mixture (MiniComplete, Roche). Immunoprecipitation of HA-synphilin1-1 was carried out with anti-HA antibody for 4 h at 4 °C. Immunoprecipitates were washed with lysis buffer containing 500 mM NaCl, and ubiquitinated synphilin-1 was detected by Western blot.

**Human Tissues**. Human substantia nigra and frontal cortex from PD and controls were obtained from Queen Square Brain Bank (UCL, London). For steady-state experiments, brain tissues were homogenized in 10 volumes of buffer used for co-immunoprecipitation experiments.

Mitochondrial preparations from PD cortices were carried out as described before [12]. Briefly, 50 mg of brain cortex was homogenized in buffer containing 250 mM sucrose, 20 mM Hepes, 3 mM EDTA, 20 mM sodium fluoride, 2 mM sodium orthovanadate, 10 mM inorganic pyrophosphate, 20 mM β-glycerol phosphate, 30 μM MG132, and a protease inhibitor cocktail (MiniComplete, Roche). Mitochondrial fractions were purified as described below in the mitochondrial preparation section.

**Mitochondrial Preparation**. Mitochondria were isolated using a discontinuous Percoll gradient, as previously described [12]. Briefly, cells or human brain tissues were homogenized in buffer containing 250 mM sucrose, 20 mM Hepes, 3 mM EDTA, 20 mM sodium fluoride, 2 mM sodium orthovanadate, 10 mM inorganic pyrophosphate, 20 mM β-glycerol phosphate, 30 μM MG132, and protease inhibitor cocktail (MiniComplete, Roche) using a glass homogenizer. Homogenates were clarified by centrifugation at 2500× *g* for 5 min. The resulting supernatant was centrifuged at 13,000× *g* for 5 min, and the crude mitochondrial pellet was washed once. The pellet was then resuspended in 15% Percoll in cold homogenizing buffer, layered over a discontinuous Percoll gradient (23% over 40%), and centrifuged at 73,000× *g* for 20 min at 4 °C. The mitochondrial fraction was collected from the 23%/40% interface, washed twice in cold buffer, resuspended, and subjected to Western blot analysis. Synphilin-1 levels in mitochondrial and cytosolic fractions were quantified by ImageMaster analysis.

**Primary Neuronal Cultures**. E18 primary cortical cultures were prepared from Sprague-Dawley rats according to the protocol approved by the committee for animal experimentation at the Technion-Israel Institute of Technology (protocol number IL0160124). Briefly, after decapitation, the cortices were retrieved and maintained in Hank’s balanced salt solution. Then, the samples were digested by incubation for ten minutes with trypsin (Beit-Haemek Biological Industries, Beit HaEmek, Israel) at 37 °C. This was followed by physical trituration with Pasteur pipettes, and the samples were briefly centrifuged for 10 s at 1000× *g* to remove debris. The supernatant was filtered through a 0.7 µm filter (BD Biosciences, San Jose, CA, USA) followed by centrifugation at 900× *g* for 4 min. The cell pellet was resuspended in Neurobasal medium supplemented with B27 (Invitrogen, Carlsbad, CA, USA) and 0.5 mM L-glutamine (Beit-Haemek Biological Industries). Neurons were plated in 12 or 6-well plates covered with poly-D-Lysine (Sigma) at a density of 0.45 and 2 × 10^6^ cells per well, respectively. Transfections were carried out at DIV12 using the calcium phosphate method. After 72 h, transfected neurons were processed for immunocytochemistry, as described below.

**Immunocytochemistry Assays**. HEK293 cells were processed for immunocytochemistry as before [12]. Briefly, cells were fixed in 4% paraformaldehyde for 15 min and permeabilized with PBS containing 0.2% Triton X-100. Cells were blocked in PBS containing 0.1% Triton X-100 and 5% normal goat serum for 1 h and incubated with primary antibodies for an additional hour. Immunolabeling was visualized using FITC- and Cy^3^-labeled secondary antibodies (1 μg/mL; Jackson Laboratories, Bar Harbor, ME, USA).

For mitophagy experiments, a small amount of GFP was co-transfected to identify transfected cells, and mitochondrial content was assessed by staining the endogenous mitochondrial matrix protein HSP60 with anti-HSP60 antibodies. Cells were processed 36 h post-transfection and analyzed by confocal microscopy (Zeiss LSM 880, Jena, Germany) to determine the percentage of cells lacking mitochondria. For each field, 1 μm optical sections were acquired at three different focal planes using identical laser settings and magnification. GFP-positive cells were evaluated for the presence or absence of mitochondria (HSP60 signal) across all three focal planes to ensure accurate detection and avoid missing any mitochondria. The images shown represent the best focal plane of a chosen field. Approximately 200 cells were examined for each condition in every independent experiment, and the data are representative of at least three independent experiments.

**Statistics**. Statistical analysis was performed by repeated measures one-way ANOVA with Bonferroni’s multiple comparison test or two-tailed Student’s *t*-test using GraphPad Prism software version 6.03 (GraphPad Inc., San Diego, CA, USA). Differences were considered significant when *p* ≤ 0.05. All Western blots shown in this study are representative of at least three independent experiments.

## Data Availability

Data sharing is not applicable to this article as no datasets were generated or analysed during the current study.

## Supplementary Materials

The following supporting information can be downloaded at: https://www.mdpi.com/article/10.3390/ijms26168048/s1.

## Abbreviations

CDK5, cyclin dependent kinase 5; HA, hemagglutinin; PD, Parkinson’s disease; SIAH, Seven in Absentia Homolog; siRNA, short interfering RNA.
