# Supplementary material for: CDK5 Inhibits Synphilin-1 Ubiquitination and Basal Mitophagy: Implications for Parkinson’s Disease
Source: Int J Mol Sci. 2025 Aug 20;26(16):8048. doi: 10.3390/ijms26168048 (PMC12386666; doi:10.3390/ijms26168048)

Supplementary Information.

Figure S1. Mass Spectrometry analysis of synphilin-1. HEK293 cells were transfected with HA-synphilin-1 in the absence (**A**) and presence of His-CDK5 and His-p25 (**B**). Synphilin-1 was immunoprecipitated with anti-HA and processed for Mass Spectrometry analysis. Both panels A and B show the full-length amino acid sequence of synphilin-1. The yellow highlighted areas represent the amino acid stretches sequenced by MS, while the green ones represent the identified post-translationally modified residues. Green highlighted S and T represent phosphorylated residues.

A

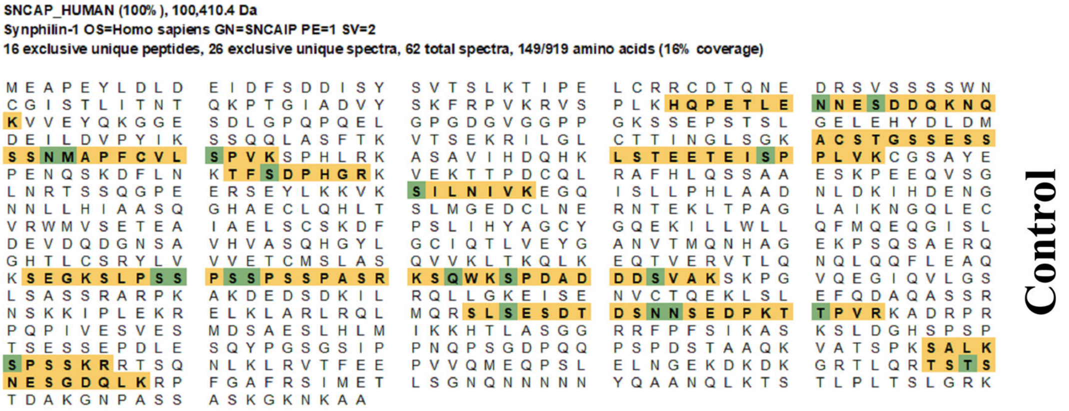

B

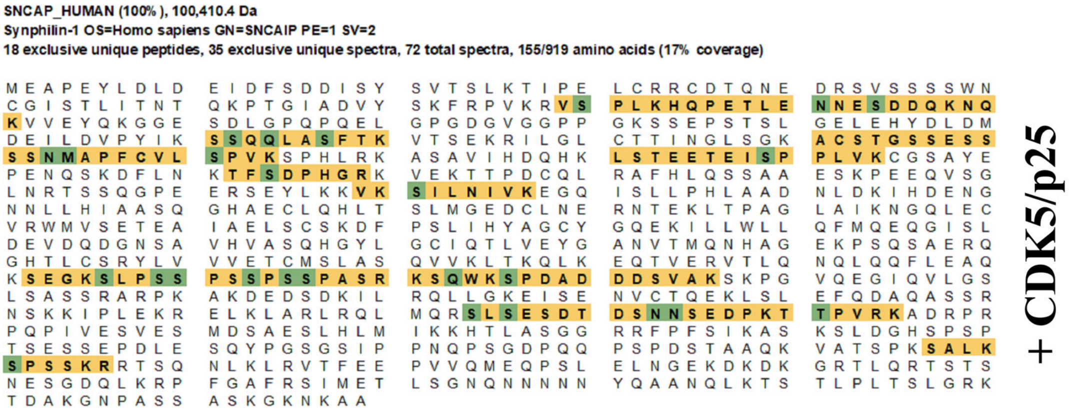

Supplement: Supplementary file 1 [file ijms-26-08048-s001.zip › Supplementary Information CDK5 MS 25.pdf]
